# Evaluation of Health Education on Oral Cancer Screening Knowledge and Skills Among Community Health Workers in Agra District, India: A Community-Based Interventional Study

**DOI:** 10.7759/cureus.98062

**Published:** 2025-11-29

**Authors:** Shubham Ruhela, Geetu Singh, Akhil P Singh, Himalaya Singh, Purnoor Kaur

**Affiliations:** 1 Preventive Medicine, Sarojini Naidu Medical College, Agra, IND; 2 Community Medicine, Sarojini Naidu Medical College, Agra, IND; 3 Otolaryngology - Head and Neck Surgery, Sarojini Naidu Medical College, Agra, IND

**Keywords:** community health workers, health education, oral cancer, oral visual examination, screening

## Abstract

Objective: To evaluate the effect of health education intervention on the knowledge and skills of community health workers (CHWs) regarding oral cancer screening in the Agra district, India.

Methods: A community-based interventional study on oral cancer was commissioned among community healthcare workers in the Agra district. A total of 242 CHWs participated, selected via purposive sampling. Data was collected using a pre-tested, semi-structured questionnaire. A 45-minute educational session with audiovisual aids and hands-on oral visual examination (OVE) training was delivered. Post-intervention assessment was done after one month. Statistical analysis included paired t-tests and chi-square tests (p < 0.05 considered significant).

Results: The mean knowledge score of CHWs increased from 26.6 ± 8.36 to 39.3 ± 7.51 (47.74% improvement; p < 0.001). Skill scores improved from 4.79 ± 2.82 to 8.18 ± 3.68 (70.77% improvement; p < 0.001). The proportion of CHWs with good knowledge rose from 4.5% to 28.5%, and those with poor knowledge declined from 69.8% to 13.2%.

Conclusion: Targeted training interventions can significantly improve CHWs’ competence in oral cancer screening. Scaling up such initiatives and integrating them into national non-communicable disease (NCD) programs can strengthen early detection efforts in resource-limited settings.

## Introduction

Oral cancer, which includes malignancies of the lips, various parts of the mouth, and the oropharynx, ranks as the 13th most diagnosed cancer across the globe. In 2022 alone, approximately 389,485 new cases of oral and lip cancer were reported worldwide, resulting in around 188,230 deaths [1]. As per the Global Cancer Observatory (GLOBOCAN) 2022 data, India recorded approximately 137,000 new cases of oral cancer and more than 78,000 deaths linked to the disease. These figures point to a concerningly high mortality rate, highlighting both the aggressive nature of the condition and the challenges in early detection and treatment [1,2]. Data trends show oral cancer affects men more frequently and is generally more fatal in males compared to females [1]. The risk also increases with age and is significantly influenced by socio-economic factors. India contributes to nearly one-third of all oral cancer cases globally [3,4].

According to the Global Adult Tobacco Survey (GATS) 2016-2017, overall tobacco use in India declined from 34.6% in 2009-2010 (GATS-1) to 28.6% in GATS-2. Currently, 28.6% of adults use tobacco in some form - 10.7% smoke and 21.4% use smokeless tobacco. Men show higher usage rates than women. Uttar Pradesh reports a higher prevalence than the national average, with 13.7% smoking and 29.4% using smokeless tobacco. These regional disparities emphasize the need for focused tobacco control strategies in high-burden states [5].

Oral cancer poses a significant public health challenge in India, primarily due to delayed diagnosis, which results in poor outcomes and high treatment costs [6]. Oral visual examination (OVE) is a simple, non-invasive screening technique that facilitates early detection, enhancing treatment effectiveness and lowering mortality. The Kerala Oral Cancer Screening Trial demonstrated that four rounds of OVE conducted by trained community health workers (CHWs) led to an 81% reduction in mortality and a 38% decrease in incidence. As an affordable method, OVE is well-suited for low-resource settings and improving access among underserved communities [7].

In addition to accredited social health activists (ASHAs), auxiliary nurse and midwives (ANMs) and community health officers (CHOs) also conduct cancer screenings. Individuals with positive results are referred to the nearest health and wellness centre (HWC), where CHOs assess them using an OVE kit. Suspected cancer cases are further referred to primary health centres (PHCs), community health centres (CHCs), or district hospitals as per protocol. By June 2021, 76,877 HWCs were set up, and around 57.86 million people had been screened for oral cancer [8].

CHWs often struggle to motivate participation in oral cancer screening due to limited public awareness of oral premalignant disorders (OPMDs) and their cancer risk [9]. Though seemingly harmless, OPMDs have an 8% malignant transformation rate, potentially progressing over 1-20 years [10]. To address this, decentralized screening via OVE is promoted, especially in resource-limited areas, to improve early detection and reach high-risk, underserved populations [11].

The National Programme for Prevention and Control of Non-Communicable Diseases (NP-NCD) recommends that adults between the ages of 30 and 65 undergo opportunistic health evaluations (OVE) every five years to aid with the early detection and management of these chronic conditions [12].

Oral cancer screening, though reflective of systemic health, is often overlooked. Training of CHWs, especially ASHAs, in oral examination and lesion detection can enhance early diagnosis. Integrating screening into primary care services and empowering CHWs with the necessary skills strengthens rural health systems and promotes community awareness [13].

The survey also highlights a concerning lack of awareness and participation in oral cancer screening. In India, only 0.9% of women have ever been screened for oral cancer, and this rate drops to just 0.6% in Uttar Pradesh. For men, the national screening rate stands at 1.2%, while in Uttar Pradesh it is marginally lower at 1.1%. These numbers reflect a significant gap in preventive healthcare practices, especially in high-risk states like Uttar Pradesh [14]. This study aims to evaluate the effect of a health education intervention on the knowledge and skills related to oral cancer screening among CHWs in the Agra district.

## Materials and methods

Study design and setting

A community-based interventional study was conducted between December 2022 and December 2024 in three selected blocks of Agra: Barauli Ahir, Bichpuri, and Khandauli, for operational feasibility and geographical proximity. The district comprises both urban and rural regions and includes 16 community development blocks. This study was approved by the Institutional Ethical Committee, Sarojini Naidu Medical College, Agra, vide no. SNMC/IEC/THESIS/2023/185.

Study population

The study targeted CHWs, including ASHAs, ANMs, and CHOs working in rural and urban areas of Agra district. The only eligibility criteria for inclusion were a minimum of six months of field experience and consent to participate. No exclusion criteria were used.

Sample size and sampling technique

A total of 257 CHWs were initially enrolled using purposive sampling. Of these, 242 participants completed both the pre- and post-intervention assessments and were included in the final analysis. Participants were recruited from three CHCs, where monthly review meetings of CHWs are routinely held. The purposive sampling technique has been opted for in the present study. All CHWs (ASHAs, ANMs, and CHOs) found at selected blocks during the study period were included in this study.

Study tool

Data were collected using a pre-tested, semi-structured, self-administered questionnaire developed by the authors with some adaptation from the National Health Mission (NHM) training module for Multi-Purpose Workers on NCD prevention and screening [15]. The questionnaire was translated into Hindi using forward and backward translation for conceptual accuracy. It included 12 questions assessing knowledge about oral cancer, 12 multiple-response questions on awareness and screening practices, 14 questions assessing practical skills in OVE, and items on perceived barriers and facilitators of screening. Expert feedback from specialists in ENT and community medicine was incorporated.

Intervention

All participants first completed a baseline (pre-test) questionnaire assessing knowledge and skills related to oral cancer and screening. This was followed by a structured 45-minute health education session by the first author delivered to a group of 20-22 CHWs over 12 sessions. The session included interactive lectures, audiovisual content, posters, flipcharts, and hands-on training in OVE using dummy models. All content was tailored to participants' educational backgrounds and conducted in Hindi for better comprehension. A post-test using the same questionnaire was conducted one month after the intervention to assess changes in knowledge and skills.

Scoring and categorization

Responses were scored with 1 mark for each correct answer. For knowledge and skills, percentage scores were calculated, and participants were graded as poor if the score was <50%, average for 50-75%, and good for >75%.

Statistical analysis

Data were entered and analyzed using Jamovi software (version 2.6.25; Jamovi Project, Sydney, Australia). Descriptive statistics (mean, standard deviation, frequency, and percentages) summarized the socio-demographic characteristics and test scores. Paired t-tests were applied to compare mean pre- and post-intervention scores. Chi-square tests were used to analyze categorical variables. A p-value < 0.05 was considered statistically significant. The percentage change in individual scores was calculated using pre- and post-test scores.

## Results

The sociodemographic characteristics of the study participants are as mentioned in Table 1. The females dominate the workforce of CHWs. The mean ages of ASHA, ANM, and CHO were 38.64, 35.24, and 31.19 years, respectively.

**Table 1:** Sociodemographic profile of the participants (N = 242) ASHA: accredited social health activist; ANM: auxiliary nurse and midwife; CHO: community health officer

Variable	Study participants		
Age group (in years)	ASHA, N (%)	ANM, N (%)	CHO, N (%)
<25	6 (4.05)	2 (4.25)	9 (19.1)
26-35	50 (33.7)	25 (53.1)	29 (61.7)
36-45	54 (36.4)	16 (34.0)	5 (10.6)
46-50	29 (19.5)	2 (4.25)	3 (6.38)
>50	9 (6.08)	2 (4.25)	1 (2.12)
Total	148 (100.0)	47 (100.0)	47 (100.0)
Gender			
Female	148 (100.0)	47 (100.0)	13 (27.6)
Male	0 (0.0)	0 (0.0)	34 (72.3)
Total	148 (100.0)	47 (100.0)	47 (19.4)
Religion			
Hindu	145 (97.9)	47 (100.0)	41 (87.2)
Muslim	3 (2.03)	0(0.0)	5 (10.6)
Christian	0(0.0)	0(0.0)	1 (2.12)
Total	148 (100.0)	47 (100.0)	47 (100.0)
Marital status			
Married	138 (93.2)	44 (93.6)	30 (63.8)
Unmarried	0(0.0)	3 (6.38)	17 (36.1)
Widow	10 (6.75)	0(0.0)	0(0.0)
Total	148 (100.0)	47 (100.0)	47 (100.0)
Education			
Up to 8th Class	42 (28.3)	0(0.0)	0(0.0)
High School	47 (31.7)	0(0.0)	0(0.0)
Intermediate	46 (31.0)	13 (27.6)	0(0.0)
Graduate	9 (6.08)	17 (36.1)	44 (93.6)
Postgraduate	4(2.70)	17 (36.1)	3(6.38)
Total	148 (100.0)	47 (100.0)	47 (100.0)

Before the training, there were 6.2% of the CHWs had never heard of oral cancer. Additionally, two-thirds of the CHWs believed that prevention of oral cancer is possible; post-intervention, this proportion rose to 95.5%. Similarly, only 55% seemed to think that treatment of oral cancer is possible; expectedly, this proportion also rose to 90.5%. Furthermore, the understanding that oral cancer is not contagious increased from 44.6% to 57.4%. The intervention was also effective in enhancing the proportion of CHWs who knew early detection leads to a better prognosis, with awareness rising from 53.7% to 90.9%. In line with this, knowledge about potential signs of oral cancer, like recognition of white or red patches, also showed significant improvement, from 50.4% to 85.5%. Another misconception was cleared for a considerable proportion of CHWs: that oral cancer cannot be prevented through vaccination (35.1% to 60.3%).

The knowledge of CHWs regarding oral cancer significantly improved following the health education intervention. The most notable improvement was in the category of signs and symptoms, with a 132.6% increase (from 27.0% to 62.8%), followed by harmful effects and risk factors, which rose by 67.5% (from 36.9% to 61.8%). Moderate gains were observed in treatment and referral (35.3% increase, from 44.8% to 60.6%), general awareness (34.6% increase, from 56.7% to 76.3%), and prevention (28.1% increase, from 42.7% to 54.7%). Overall, the knowledge level improved from 41.6% to 63.2%, reflecting a 47.74% increase following the intervention. This finding was statistically significant (Table 2).

**Table 2:** Pre- and post-intervention knowledge levels about oral cancer among study participants (N = 242) Student’s t-test was performed, with a threshold for statistical significance set at p < 0.05.

Category	Pre-test (%)	Post-test (%)	Improvement (%)	t-value	p-value
Signs and symptoms	27	62.8	132.59	27.75	<0.001
Harmful effects/risk factors	36.9	61.8	67.46	21.47	<0.001
Treatment and referral	44.8	60.6	35.27	16.46	<0.001
General awareness	56.7	76.3	34.57	20.42	<0.001
Prevention	42.7	54.7	28.10	15.58	<0.001
Overall improvement	41.6	63.2	47.74	27.67	<0.001

According to their pre- and post-intervention knowledge scores on oral cancer, CHWs - ASHA, ANM, and CHO - were categorized as possessing "good" or "bad" knowledge. In the pre-test, the majority of ASHAs (83.8%) and ANMs (78.7%) were in the poor knowledge category, compared to only 17.0% of CHOs. Most CHOs (61.7%) were in the average category, and 21.3% were in the good category, showing a higher baseline knowledge level. Following the intervention, there was a notable improvement across all groups. The percentage of ASHAs in the poor category decreased significantly from 83.8% to 18.2%, while 22.3% moved into the good category. Among ANMs, good knowledge levels increased dramatically from 2.1% to 48.9%. CHOs continued to perform well, with no participants in the poor category post-test and 27.7% in the good category (Table 3).

**Table 3:** Grading of community health workers according to their pre- and post-intervention knowledge level participating in oral cancer (N = 242) Student’s t-test was performed, with a threshold for statistical significance set at p < 0.05. ANM: auxiliary nurse and midwife; CHO: community health officer

Test	Grading	ANM	CHO	Chi-square test
		N (%)	N (%)	
Pre-test	Poor	37 (78.7)	8 (17.0)	χ^2^ = 89.5; p < 0.001
	Average	9 (19.1)	29 (61.7)	
	Good	1 (2.1)	10 (21.3)	
	Total	47 (100.0)	47 (100.0)	
Post-test	Poor	5 (10.6)	0 (0.0)	χ^2^ = 22.4; p < 0.001
	Average	19 (40.4)	34 (72.3)	
	Good	23 (48.9)	13 (27.7)	
	Total	47 (100.0)	47 (100.0)	

The oral cancer knowledge levels of CHWs significantly improved following the intervention, as evidenced by the results of the paired t-test. Specifically, 57% of ASHAs and 59% of ANMs showed significant improvement in their pre- and post-test scores. CHOs had higher baseline knowledge levels compared to other groups, and they also demonstrated a statistically significant increase after the intervention. Overall, the mean knowledge score improved from 26.6 ± 8.36 in the pre-test to 39.3 ± 7.51 in the post-test, reflecting a 47.74% increase. This change was statistically significant, with a p-value of < 0.001 (Table 4).

**Table 4:** Comparative analysis of overall knowledge score of various CHWs A p-value < 0.05 was considered statistically significant. ASHA: accredited social health activist; ANM: auxiliary nurse and midwife; CHO: community health officer; CHWs: community health workers

Designation	Pre-test, mean ± SD	Post-test, mean ± SD	Paired t-test	Improvement (%)
ASHA	23.9 ± 5.49	37.5 ± 7.57	p < 0.001	57
ANM	26.6 ± 6.47	42.2 ± 8.61	p < 0.001	59
CHO	36.3 ± 10.13	42.0 ± 3.38	p < 0.001	16
Overall knowledge	26.6 ± 8.36	39.3 ± 7.51	p < 0.001	47.74

With regard to the distribution of CHWs according to their overall skill level scores before and after the oral cancer screening intervention, the mean pre-test score was 4.79 ± 2.82, with scores ranging from 0 to 16. Following the intervention, the mean post-test score increased to 8.18 ± 3.68, with a range of 2 to 16. The paired t-test value was 9.88, with a p-value of <0.0001, indicating a highly significant improvement. The absolute difference in mean scores was 3.39, corresponding to a 70.77% improvement in overall skills after the intervention (Table 5). Appendices A-F were used for data collection.

**Table 5:** Skill level scores: pre-test vs. post-test Student’s t-test was performed, with a threshold for statistical significance set at p < 0.05.

Skills level score	Test	Mean ± SD	Paired t-value	Absolute difference	Improvement (%)
						Pre-test	4.79 ± 2.82	9.88	3.39	70.77
	Post-test	8.18 ± 3.68	<0.0001							

**Figure 1:**
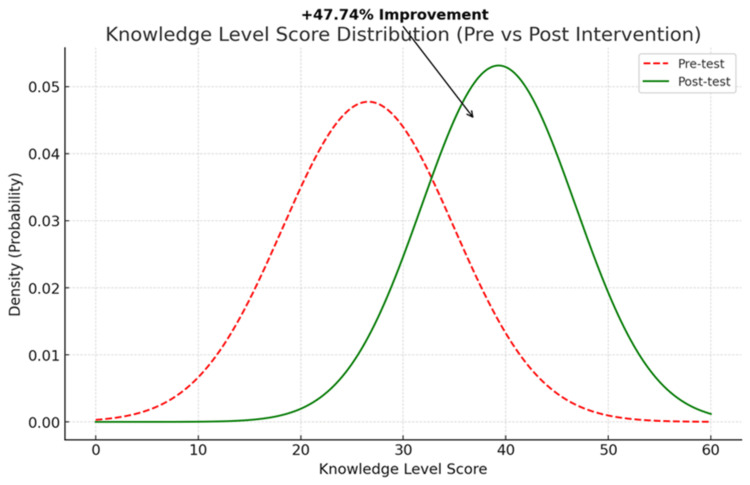
Distribution curve of knowledge-level scores among community health workers before and after the intervention.

The overall mean knowledge scores of CHWs regarding oral cancer improved following the intervention. A comparison of mean scores revealed that the pre-test mean was 26.6, while the post-test mean increased to 39.3, reflecting a 47.74% improvement in knowledge related to oral cancer screening. This improvement was found to be statistically significant (p < 0.001) (Figure 1).

## Discussion

Oral cancer is a major public health concern in India, particularly due to late detection, which leads to poor treatment outcomes and high costs [6]. We evaluated the effect of health education interventions by comparing pre- and post-test knowledge and skill scores on oral cancer prevention, risk factors, signs, symptoms, treatment, and screening techniques. The main findings of the study revealed that initially, the majority of ASHAs (83.8%) and ANMs (78.7%) had poor knowledge. However, after the health intervention, the proportion of ASHAs, ANMs, and CHOs with poor knowledge dropped significantly to 18.2%, 10.6%, and 17.0%, respectively.

Our analysis reported that ASHA workers and ANMs predominantly belonged to the age group of 36-45 years old, while CHOs were mostly in the age group of 26-35 years old. These study findings align with the studies conducted by Birur et al. (2020), which showed that most of the participants (ASHA) belong to the age of 35 years, and Bhagia et al. (2020), which showed that most of the participants (ASHA and ANM) belong to the age group of 36-50 years old [16,17]. Sandhya et al. (2014) found that most of the CHWs belong to the age group of 20-30 years. Rao et al. (2023) and Mubin et al. (2021) reported that most of the CHWs were 31-40 years of age. Meenu et al. (2025) and Fotedar et al. (2018) found the age group of the study participants to be between 40 and 50 years [18-22]. These variations in age groups of ASHAs may be due to community recruitment of ASHAs, where usually elder participants are more inclined to join due to lower family responsibilities, different geographical regions, and varying speeds of implementation of the National Rural Health Mission (NRHM) program across states. In contrast, ANMs and CHOs are employed directly within the government healthcare system immediately after completing their qualifications; hence, they are usually younger in age groups [19,23,24].

Our findings showed that all the ASHAs and ANMs were females; ASHAs are educated to the high school level, and most ANMs and CHOs have a graduate level of education. This was similar to other studies conducted by Fotedar et al. (2018), Bhagia et al., Birur et al. (2020), and Rao et al. (2023) [16,17,19,22]. The female ASHA health workers work on an incentive basis and are viewed as link workers or facilitators, providing effective, efficient, and affordable health care within the community primarily for maternal and child health [19]. Another study from Andhra Pradesh and Kerala found that most of the CHWs had secondary school education [18,21]. The Committee on the Empowerment of Women, Ministry of Health & Family Welfare, in the 11th Report of the 15th Lok Sabha, recommended raising the educational qualification for ASHAs to a minimum of 10th standard [25-27]. Favorably, our study participants met the suggested educational requirements for ASHAs and retained significant knowledge from the training session.

Our interventional analysis found significant improvement (27% to 62.8%) in knowledge about the signs and symptoms of oral cancer. Our finding is consistent with Sandhya et al. (2014), who found that correct responses on signs and symptoms of oral cancer of the study participants increased from 17% in the pre-test to 53% in the post-test [18]. We also found significant improvement in knowledge regarding treatment options, consequences of incomplete treatment, and referral for oral cancer. Das et al. (2014) showed that repeated training can enhance the early detection of infection cases and referral to PHC for treatment [25]. Repeated training of the ASHAs has shown an increased knowledge and referral rate. Similarly, Saprii et al. (2015) demonstrated that integrating oral health education and training for ASHAs can enhance awareness of oral health issues at the village level, supporting our point [24]. Studies from Kerala and Karnataka reported better scores of knowledge on oral cancer, and the plausible reason for better responses to questions in the domains of knowledge of oral cancer is that CHWs work as facilitators in providing basic medical care in their community and are in regular contact with nurses and doctors in these regions [19,25,26,28].

Furthermore, in our study, CHOs performed better than ASHAs and ANMs in both pre- and post-tests. However, ANMs showed the most improvement, indicating a strong impact of the intervention. A probable reason for this variance could be the contribution of ANMs with 5-10 years of experience, adding to the effectiveness of the intervention. Additionally, those with graduate and postgraduate qualifications, along with CHOs holding postgraduate education, performed better, further enhancing their knowledge scores. This highlights the need for CHWs' capacity building, which is important, as their knowledge level needs to be improved further for better services and health outcomes [26].

There could be several reasons for the significant improvement in their knowledge level as assessed after one month of intervention. Firstly, the combination method, which includes posters, pamphlets, and health talks, is usually the most effective approach for educating CHWs. Secondly, hands-on training, which was provided to a small group of CHWs, helped them easily identify oral lesions. Alongside the site-specific oral examinations and their connection to the location of quid placement, CHWs received in-depth training on tobacco-associated mucosal lesions as white, red, and mixed patches on the oral mucosa. Furthermore, the anatomical ease of accessing the oral cavity for examination in general allows them to effectively detect mucosal alterations. Additionally, the up to 20 years of fieldwork experience of ASHAs, along with intermediate education levels, make them suitable candidates for new screening skills due to the confidence that the community bestows upon them. A study by Sankaranarayanan (1997) suggests that the integration of oral examination, along with other responsibilities assigned to the ASHAs, would improve the awareness of oral cancer [29].

Basic oral cancer screening clinical skills were assessed before and after intervention on 94 CHWs (ANMs and CHOs), with the majority showing significant improvement. Since ASHA workers aren't expected to perform clinical examinations, they were excluded from the analysis. Notably, the ability to identify tobacco users who needed monthly self-examinations increased from 69.1% to 92.6%. Thampi et al. (2022) reported comparable results, with 89% showing comparable improvements [30]. According to Patil et al. (2019), the oral cavity is easily accessible, and oral cancers are often preceded by disorders or lesions that can be detected early during routine check-ups or screenings by health workers [23].

Our present study found a 70.77% improvement in overall skills related to oral cancer screening among CHWs one month after the intervention. Birur et al. (2020) [16] conducted a study where ASHAs participated in a three-day oral cancer screening training, followed by a three-month follow-up. The intervention included digital data capture, hands-on clinical training, and field practice. The results showed an 84% improvement in skills immediately after the training, with a 64% retention rate at the three-month follow-up.

This study has several limitations that should be considered while interpreting the findings. First, the intervention was conducted in only three blocks of Agra, which may limit the generalizability of the results to other blocks or districts with different demographic, cultural, or healthcare-access characteristics. Second, the sample size of 242 participants, though adequate for preliminary evaluation, may not fully capture the variability within the wider population. Third, the pre-post study design lacked a control group, making it difficult to attribute the observed changes solely to the intervention, as external influences and temporal factors could also have contributed. Additionally, participant responses were self-reported, raising the possibility of recall bias and social desirability bias. Finally, the relatively short follow-up period limits conclusions regarding the long-term retention of knowledge and sustained behavior change. Also, validity testing of the questionnaire was not performed in the study.

## Conclusions

Our community-based health education interventional analysis on CHWs regarding oral cancer found that the mean knowledge score of CHWs improved significantly. Skill scores also improved. It can be concluded that the targeted training interventions can significantly improve CHWs’ competence in oral cancer screening. Scaling up such initiatives and integrating them into national NCD programs can strengthen early detection efforts in resource-limited settings.

## Appendices

Appendix A

Name of respondent:                                                                         

Post of health worker:

Address:                                                                                             

Phone number:

**Table 6:** Sociodemographic details

S. no.	Particulares	Categories	Code
1)	Age (in years)	...	
2)	Gender	1. Male ( ) 2. Female ( )	
3)	What is your marital status?	1. Unmarried ( ) 2. Married ( ) 3. Widow ( ) 4. Divorced/Separated ( )	
4)	What is your education?	1. Up to 8th Class ( ) 2. High school ( ) 3. Intermediate ( ) 4. Graduate ( ) 5. postgraduate ( )	
5)	What is your religion?	1. Hindu ( ) 2. Muslim ( ) 3. Sikh ( ) 4. Christian ( ) 5. Other ( )	
6)	Where do you work? (Area)	1. Rural ( ) 2. Urban ( ) 3. Semi-Urban ( )	
7)	What is your designation?	1. ASHA ( ) 2. ANM ( ) 3. CHO ( ) 4. USHA ( ) 5. Other	
8)	How long have you been working as community health workers (ASHA, ANM, CHO)?	1. Less than 5 years ( ) 2. 5-10 years ( ) 3. 11-15 years ( ) 4. 16-20 years ( ) 5. >20 years ( )	
9)	Are you satisfied with your work/job?	1. Yes ( ) 2. No ( )	
10)	If yes/no, why?	….	
11)	What is the total number of members in your family?	….	
12)	Type of family	1. Joint family ( ) 2. Nuclear family ( ) 3. Three-generation family	
13)	What is your total family income per month?	….	
14)	Socioeconomic status as per BG prasad Scale 2023	1. Class I ≥ 8763 ( ) 2. Class II 4381.5-8675.3 ( ) 3. Class III 2630-4294 4. Class IV 1314.5-2541.27 ( ) 5. Class V <1314 ( )	
15)	Do you smoke, such as cigarettes/bidis?	1. Never consumed ( ). 2. Current user ( ) 3. Every user ( ) 4. Past user ( )	
16)	Do you consume tobacco such as gutka, khaini, or gul manjan?	1. Never consumed ( ). 2. Current user ( ) 3. Every user ( ) 4. Past user ( )	
17)	Do you have any cancer patients in your family?	1. Yes ( ) 2. No ( )	

Appendix B 

**Table 7:** Information about services provided by ASHA, ANM, and CHO

S. no.	Question	Categories	Code
1	Population of the area you cover		
2	Have you heard about the completion of the community-based assessment checklist (CBAC FORM)?	1. Yes ( ) 2. No ( )	
3	Do you encounter problems while filling out the community-based assessment checklist (CBAC FORM) completely?	1. Yes ( ) 2. No ( )	
4	Have you ever received training on NCD Program Training/Cancer/Oral Cancer Screening?	1. Yes ( ) 2. No ( )	
5	If yes, where were you trained?	...	
6	When did you get training?	...	
7	Who gave the training?	...	
8	Are you doing oral cancer screening?	1. Yes ( ) 2. No ( )	
9	Are you satisfied with doing oral cancer screening?	1. Yes ( ) 2. No ( )	
10	Give reason for satisfaction or dissatisfaction	...	
11	How many suspicious oral lesions have you identified?	...	
12	How many people have you referred for oral lesions?	...	
13	Do you educate people on oral cancer screening/tobacco during fieldwork?	1. Yes ( ) 2. No ( )	
14	Do you follow up on an oral cancer screening patient once identified during fieldwork?	1. Yes ( ) 2. No ( )	

Appendix C 

**Table 8:** Questions about knowledge and awareness about oral cancer

S. no.	Question	Categories	Code
1	Have you heard of oral cancer?	1. Yes ( ) 2. No ( )	
2	Is prevention of oral cancer possible?	1. Yes ( ) 2. No ( )	
3	Is treatment of oral cancer possible?	1. Yes ( ) 2. No ( )	
4	Is oral cancer contagious?	1. Yes ( ) 2. No ( )	
5	Does risk of oral cancer increase with age?	1. Yes ( ) 2. No ( )	
6	Does early detection of oral cancer means better prognosis?	1. Yes ( ) 2. No ( )	
7	Is a white or red patch in the oral cavity a possible initial sign of cancer?	1. Yes ( ) 2. No ( )	
8	Can cancer be completely cured?	1. Yes ( ) 2. No ( )	
9	Can cancer be prevented through vaccination?	1. Yes ( ) 2. No ( )	
10	Is cancer a hereditary disease?	1. Yes ( ) 2. No ( )	
11	What is the ideal age to start screening individuals for oral cancer?	1. Men/women of 20 years	
		2. Men/women of 30 years	
		3. Men/women of 40 years	
		4. Men/women of 50 years	
12	How often do you perform an oral visual examination of an apparently healthy individual aged above 30 years?	1. Once a week	
		2. Once a month	
		3. Once in 6 months	
		4. Once a year	
		5. Once in 5 years	
		6. Never	
13	Who, do you think, should be encouraged for oral cancer screening more often? You can select multiple answers	1. Alcohol intake	
		2. Tobacco consumption in any form	
		3. Other	
		5. I Don’t know	
14	Is there any oral cancer screening program in government hospitals that is free of cost?	1. Yes ( ) 2. No ( )	
15	Do you know about the national program NP-NCD?	1. Yes ( ) 2. No ( ) 3. There is no such government program ( )	
16	How can oral cancer be prevented? You can select multiple answers	1. Screening	
		2. Healthy eating	
		3. Vaccination	
		4. Abstinence from Alcohol and Tobacco	
		5. Regular physical exercise	
		6. Being inactive	
		7. Abstinence from consanguineous marriage	
		8. Hand washing	
		9. Body hygiene	
		10. Oral hygiene	
		11. Avoiding crowded places	
17	What are the harmful effects of smoking? You can select multiple answers	1. Increased risk of gum diseases.	
		2. White patches inside the mouth.	
		3. Tooth loss.	
		4. Oral cancer.	
		5. Lung cancer.	
		6. Others.	
18	What is/are the risk factor(s) for oral cancer? You can select multiple answers.	1. Poor oral hygiene	
		2. Oral cancers are twice as common in men as compared to women.	
		3. Unhealthy diet—low intake fresh fruits and vegetables	
		4. Family history	
		5. Tobacco use	
		6. Alcohol consumption	
		7. Betel nuts and other forms of chewing tobacco	
		8. Sharp teeth and ill-fitting dentures	
19	What are the common signs and symptoms of oral cancer? You can select multiple answers.	1. A white/red patch in the oral cavity.	
		2. Ulceration/roughened areas in the oral cavity.	
		3. Whiteness of the lining of the oral cavity.	
		4. Difficulty in tolerating spicy foods.	
		5. Difficulty in opening mouth.	
		6. Difficulty in protruding the tongue.	
		7. Change in voice (nasal voice).	
		8. Excessive salivation.	
		9. Difficulty in chewing/swallowing/speaking.	
20	What is/are the treatment option(s) for oral cancer? You can select multiple answers.	1. Surgery.	
		2. Medicines.	
		3. Radiotherapy.	
		4. Others.	
21	What are the consequences of incomplete treatment for an oral cancer patient?	1. Patient status will remain unchanged.	
		2. Patient status will improve.	
		3. Patient status will deteriorate.	
		4. Others.	
22	Where would you refer a patient if oral cancer is suspected?	1. Community Health Officer	
		2. Medical officer at PHC or CHC	
		3. Medical college	
		4. Others	

Appendix D 

**Table 9:** Information about skills of community health work for oral cancer screening

S. no.	Question	Categories	Code
1	How frequently should all habitual tobacco users do self-examination of the oral cavity	1. Monthly	
		2. Yearly	
		3. I don’t know	
2	What equipment is needed for oral cancer screening?	1. Gloves	
		2. Torch	
		3. Wooden spatula	
3	Can you describe the step-by-step process you would follow when conducting an oral visual examination for oral cancer screening?	…	
	Are you examining and looking for any swelling, growth, ulcerations, scars, sinus, or fistula over the face and neck region on the outer side during the oral examination?	1. Yes ( ) 2. No ( )	
	Do you examine the border of both the lips (lip line) with lips closed and also with the mouth slightly open during the oral examination?	1. Yes ( ) 2. No ( )	
	While examining a person who uses dentures, do you ask her/him to remove them and open her/his mouth wide open during the oral examination?	1. Yes ( ) 2. No ( )	
	Do you ask the person to rinse the mouth properly with water before starting the oral examination?	1. Yes ( ) 2. No ( )	
	While examining the oral cavity, do you take help from any volunteer to hold the torch?	1. Yes ( ) 2. No ( )	
	Do you assess the extent of mouth opening by asking the person to insert three fingers together (index, middle, and ring fingers) in the mouth?	1. Yes ( ) 2. No ( )	
	Do you hold the spatula/mirror in a pen grip while conducting the oral examination?	1. Yes ( ) 2. No ( )	
	Are you examining the cheek (buccal mucosa) on both sides (right/left) while conducting the oral examination?	1. Yes ( ) 2. No ( )	
	Are you examining the tongue and floor of the mouth while conducting the oral examination?	1. Yes ( ) 2. No ( )	
	Do you examine the palate (roof of mouth) while conducting the oral examination?	1. Yes ( ) 2. No ( )	
	Are you examining the temporomandibular joint while conducting the oral examination?	1. Yes ( ) 2. No ( )	
	Do you palpate the oral cavity while conducting the oral examination?	1. Yes ( ) 2. No ( )	

Appendix E 

**Table 10:** Barriers for oral cancer screening

S. no.	Question	Categories	Code
1	Are some patients hesitant to get screened for oral cancer?	1. Yes ( ) 2. No ( )	
2	Reason for hesitancy, at level of the community health worker (can mark >1 response)	1. Lack of training or knowledge	
		2. Lack of resources (Inadequate equipment or facilities)	
		3. Lack of time or Feeling overburdened by work	
		4. Insufficient incentives	
		5. Lack of motivation	
		6. Lack of support from supervisor	
		7. Language barrier	
		8. Other (please specify): _____	
3	Reason for hesitancy, at level of community (can mark >1 response)	1. Personal belief cancer is contagious (infection)	
		2. Discomfort during screening process	
		3. Lack motivation for getting screened	
		4. Fear of the word "cancer"	
		5. Refuse due to absence of any health problem	
		6. Social taboo	
		7. Other (please specify): ____	

Appendix F 

**Table 11:** Assessment for perceived facilitators for oral cancer screening

S. no.		Perceived facilitators	Code
1	At level of Community health worker (can mark >1 response)	1. Sense of saving lives	
		2. Financial incentives	
		3. Desire to improve community health	
		4. Convenience of screening during home visit	
		5. Supportive supervisors	
		6. Part of Teamwork	
		7. Other (please specify)	
2	At level of community (can mark >1 response)	1. Personality trait	
		2. Family history of cancer	
		3. Awareness about symptoms/ sign of cancers	
		4. Other (please specify)	
